# Knowledge, attitudes and practices of South Asian immigrants in developed countries regarding oral cancer: an integrative review

**DOI:** 10.1186/s12885-020-06944-9

**Published:** 2020-05-27

**Authors:** Nidhi Saraswat, Rona Pillay, Bronwyn Everett, Ajesh George

**Affiliations:** 1 Centre for Oral Health Outcomes and Research Translation (COHORT), School of Nursing and Midwifery, Western Sydney University/South Western Sydney Local Health District / Ingham Institute for Applied Medical Research, Liverpool, NSW Australia; 2 School of Nursing and Midwifery, Western Sydney University, Parramatta, NSW Australia; 3 School of Dentistry, Faculty of Medicine and Health, University of Sydney, Sydney, NSW Australia

**Keywords:** Oral cancer, South Asians, Immigrants, Knowledge, Attitudes, Practices, Integrative review

## Abstract

**Background:**

Oral cancer is a growing problem worldwide, with high incidence rates in South Asian countries. With increasing numbers of South Asian immigrants in developed countries, a possible rise in oral cancer cases is expected given the high prevalence in their source countries and the continued oral cancer risk behaviours of immigrants. The aim of this review is to synthesise existing evidence regarding knowledge, attitudes and practices of South Asian immigrants in developed countries regarding oral cancer.

**Methods:**

Five electronic databases were systematically searched to identify original, English language articles focussing on oral cancer risk knowledge, attitudes and practices of South Asian immigrants in developed countries. All studies that met the following inclusion criteria were included: conducted among South Asian immigrants in developed countries; explored at least one study outcome (knowledge or attitudes or practices); used either qualitative, quantitative or mixed methods. No restrictions were placed on the publication date, quality and setting of the study.

**Results:**

A total of 16 studies involving 4772 participants were reviewed. These studies were mainly conducted in the USA, UK, Italy and New Zealand between 1994 and 2018. Findings were categorised into themes of oral cancer knowledge, attitudes and practices. General lack of oral cancer risk knowledge (43–76%) among participants was reported. More than 50% people were found engaging in one or more oral cancer risk practices like smoking, betel quid/pan/gutka chewing. Some of the participants perceived betel quid/pan/gutka chewing habit good for their health (12–43.6%).

**Conclusion:**

This review has shown that oral cancer risk practices are prevalent among South Asian immigrants who possess limited knowledge and unfavourable attitude in this area. Culturally appropriate targeted interventions and strategies are needed to raise oral cancer awareness among South Asian communities in developed countries.

## Background

Oral cancer - a highly morbid disease which has become a serious public health concern [1]. It is defined as cancer that forms in the tissues of the oral cavity or the oropharynx [2] and often involves pain, impaired function, altered quality of life and death [3]. Oral cancer is one of the most common cancers globally [1, 4], and is estimated to have an annual incidence of approximately 300,000 cases worldwide [1, 5, 6]. In 2018, cancers of the lip and oral cavity were collectively estimated at 354,864 new cases with deaths reaching 177,384 worldwide [1].

There is a wide geographical variation in the incidence of oral cancer with the highest rates in South and South-East Asia [5, 6]. In particular, countries of South Asia such as India, Bangladesh, Pakistan, and Sri Lanka are considered high risk for oral cancer [6, 7]. According to the World Health Organisation (WHO), these countries have been estimated to contribute nearly 40% of newly diagnosed oral cancer cases worldwide [1, 8]. The oral cancer prevalence rates in these countries are almost twice global rates [5, 6].

Oral cancer is a multi-factorial disease linked with several risk factors and potential causative agents including consumption of tobacco and alcohol, betel quid chewing, human papilloma virus, syphilis, candidiasis, dietary deficiency, and dental trauma [4, 9, 10]. The predominance of oral cancer in South Asia is mainly attributed to the use of tobacco products like bidis, smokeless tobacco, and culturally embedded use of areca nut which is utilised in different commercial preparations [3, 9, 11]. The areca nut, is the dried seed of *Areca catechu*, often mistakenly referred to as the betel nut as it is commonly chewed along with the *Piper betel* leaf [12]. Chronic use of areca nut (with or without tobacco) in South Asian countries is based on several foundation concepts like social acceptability, religious beliefs and perceived advantages [3, 13]. However, areca nut is believed to be one of the most commonly consumed psychoactive substance [14] and has been shown to have carcinogenic potential which increases when mixed with tobacco [9]. Furthermore, the practice of areca nut chewing in any form often leads to addiction and may persist as a lifelong habit [13].

People from Afghanistan, Bangladesh, Bhutan, India, the Maldives, Nepal, Pakistan and Sri Lanka (collectively known as South Asians) comprise one quarter of the world’s population and are one of the fastest growing ethnic groups in many developed countries including the United States of America [15] Canada [16], the United Kingdom [17] and Australia [18]. For several years India has been the largest source of international migrants among South Asian countries, with 17 million migrating in 2017 [19]. Bangladesh (7 million) and Pakistan (6 million) ranked 5th and 7th respectively in terms of largest country of origin of international migrants [19].

With increasing South Asian immigrants in developed countries, a possible rise in oral cancer cases could be expected given the high prevalence in their source countries [1]. As immigrants are believed to bring with them their native cultural behaviours, practices, and beliefs [3, 13], this can modify the patterns of oral diseases in destination countries too [13]. Previous literature [13, 20–22] has described typical lifestyles of immigrants in developed countries and its relevance to oral cancer incidence in their native nations. Although several studies have explored oral cancer risk behaviours of South Asian immigrants across various developed countries [20, 21, 23–28], a synthesis of these results has not yet been conducted. Gathering this information will help to inform health service planning and the need for educational and early oral cancer risk assessments in this population.

Aim- The aim of this integrative review is to synthesise all available evidence regarding the knowledge, attitudes and practices of South Asian immigrants in relation to oral cancer in developed countries.

## Methods

This study used the Preferred Reporting Items for Systematic Reviews and Meta-analyses (PRISMA) statement [29, 30] for reporting the findings from this integrative review. The protocol for this integrative review was registered with PROSPERO-International prospective register of systematic reviews (registration ID: CRD42019121410). The decision to do an integrative review [31, 32] was taken to have potential insights into qualitative, quantitative and mixed method studies.

### Inclusion and exclusion criteria

All studies included in this review met the following inclusion criteria: 1) Peer reviewed English language publications; 2) conducted on South Asian immigrant population in developed and High-income countries; and 3) explored at least one study outcome (knowledge, attitudes or practices associated with oral cancer risk). Since very little is known in this area; qualitative, quantitative and mixed method studies were eligible for inclusion in the review. Interventional studies with a pre-intervention survey component were also included. Further, no restrictions were placed on the year of publication, quality, and setting of the study.

### Data sources and search strategy

The first author worked closely with an experienced healthcare librarian to develop the search strategy which was undertaken using a combination of key words and search terms including: “oral cancer”, “oropharyngeal cancer”, “oropharyngeal neoplasm”, “oropharyngeal tumour”, “mouth neoplasms”, “mouth cancer”, “oral tumours”, India*, Pakistan*, Nepal*, Sri Lanka*, Bangladesh*, “south Asian”, “Asian”, immig*, and “immigrants” (see Additional file 1 for search terms/strategy for databases). Databases searched included Ovid-Medline, Embase, CINAHL, Scopus, and ProQuest Central. Individual search strategies were used considering the database specific indexing terms.

The search terms were used in combination using ‘Boolean’ operators (AND/OR) and MeSH (Medical Subject Heading) terms. The filter applied in the search included language (English). In addition, another experienced university librarian was consulted to ensure the appropriateness and relevance of the individual search strategies.

A final search was carried out in April 2020 to ensure inclusion of the most recent literature in this review. The reference lists of all relevant studies were also searched for additional studies.

### Article selection and screening

**Fig. 1:**
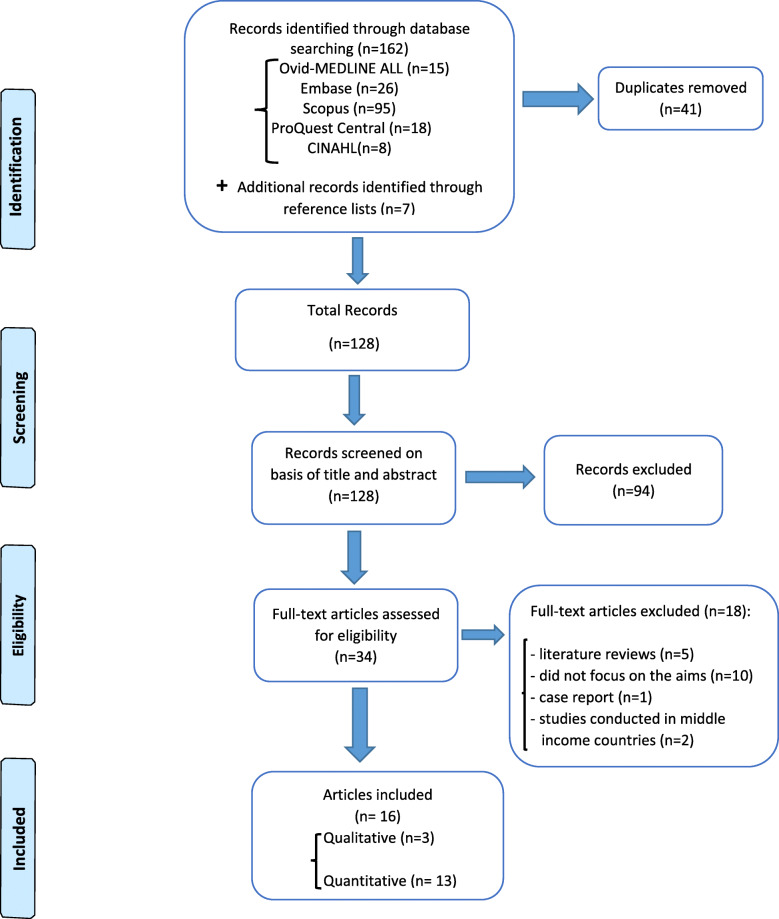
Study selection process

The search results were organised using the EndNote® bibliographic software. The title and abstract of the remaining studies were assessed by two experienced authors [NS and RP] for suitability using the inclusion and exclusion criteria. Full text articles were obtained in case of difficulty regarding decision making on the basis of title and abstract only. The full text articles were reviewed by two authors [NS and RP] independently, and then together if there was a doubt or discrepancy (see Additional file 2 for full text screening of articles). A third author [AG] was consulted to resolve any further discrepancies in judgement to assist with a final decision on inclusion or exclusion of the article. The search and selection process are illustrated in Fig. 1 (see Fig. 1 for study selection process).

### Quality assessment

The critical appraisal for selected articles was undertaken by two independent reviewers (RP and NS) to assess the methodological quality. For the quality assessment, two separate checklists were used- Critical Appraisal Skills Programme (CASP) checklist for Qualitative studies [33] and the Joanna Briggs Institute (JBI) checklist for Quantitative studies [34] (See Additional files 3 and 4). A third reviewer (AG) was consulted to reconcile any discrepancies in the quality assessments. The quality of these studies was calculated using a scoring criteria [35]. According to this criteria, score was given as a percentage (1 point for each applicable item) and the overall quality was rated as good (80–100%), fair (50–79%), and poor (< 50%) [35].

### Data extraction and synthesis

Since both the qualitative and quantitative studies were to be included in the review, the decision was made to do a narrative synthesis in line with the guidance provided by Popay et al. [36]. The aim of narrative synthesis is to “tell the story” from the findings from the included studies, whether they are qualitative, quantitative or mixed methods [36].

Subsequently, the data extraction tables were developed and piloted independently by two authors (NS and RP) and modified as required (Table 1 and Table 2)*.* The information extracted in these tables included author, year of publication, country, study characteristics and key outcomes. Data were extracted by one author (NS) and checked by two authors (RP and AG) for accuracy. A systematic review and meta-analysis of quantitative studies was not feasible due to the heterogeneity of the studies in relation to their approaches to measuring and reporting the knowledge, attitudes, and practices of South Asian immigrants regarding oral cancer risk.

**Table 1:** Study characteristics

S.No.	Author Year of publication	Country	Methodology Data collection method	Sample characteristics					Response (rate %)
				Sample size	Ethnic group	Gender (%)	Socio-economic status (as reported in study)	Age range (years)	
1	Summers et al. 1994 [37]	UK	Quantitative home-based structured interviews	296	Bangladeshi	F = 100 M = 0	Low	25–68	98.6
2	Pearson et al. 1999 [38]	UK	Quantitative Questionnaire	158	Bangladeshi	F = 42 M = 58	NR^ª^	40–83	85
3	Shetty et al. 1999 [27]	UK	Quantitative Questionnaire	367	South Asians (Indian, Pakistani, Bangladeshi etc.)- percentage not reported clearly	M = 56.1	Low-middle	16–65	NR
4	Khan et al. 2000 [39]	UK	Quantitative Questionnaire	390	Indian (21.5%), Pakistani (4.3%), Bangladeshi (9.2%)	F = 83.8 M = 16.2	Low-middle	> 16 (Median age = 44)	NR
5	Vora et al. 2000 [28]	UK	Quantitative Questionnaire	524	South Asians (Indian, Pakistani, Bangladeshi, Sri Lankan)- percentage not reported	M = 100 F = 0	Low	16–87	NR
6	Prabhu et al. 2001 [26]	UK	Quantitative Questionnaire	204	Bangladeshi	M = 51.5	Low	12–18	70.0
7	Changrani et al. 2006 [21]	USA	Pilot study (quantitative) Questionnaire	138	Indian (30.4%) Bangladeshi (69.5%)	M = 55.79	Low-middle	> 18	96
8	Croucher et al.2011 [40]	UK	Quantitative Structured interviews	369	Bangladeshi	M = F	NR	> 30	77
9	Siddique et al. 2013 [41]	UK	Quantitative Questionnaire	96	Indian-Gujarati	M = 53.1	NR	16–81	100
10	Lokhande et al. 2013 [22]	New Zealand	Qualitative Semi-structured interview	10	South Asians (Indian = 90%, Pakistani = 10%)	M = 100 F = 0	NR	18–67	NR
11	Banerjee et al. 2014 [23]	USA	Qualitative 6 Focus groups	39	Indian(38.5%), Pakistani(28.2%), Bangladeshi(33.3%)	M = 87.2 F = 12.8	NR	25–71	NR
12	Hrywna et al. 2016 [42]	USA	Qualitative 8 Focus groups	78	South Asians (Indian = 83.3%),	M = 60.3	NR	18–67	NR
13	Merchant et al. 2016 [24]	UK	Quantitative Questionnaire	201	South Asians (Indian = 77%, Pakistani = 16%, Bangladeshi, Sri Lankan, Malaysian-Indian)	M = 61	NR	18–44	NR
14	Shi et al. 2017 [43]	USA	Quantitative Questionnaire	73	South Asian (12.3%) Rest (Other countries)	M = 50.7 F = 49.3	NR	37.67	54.8
15	Mukherjea et al. 2018 [44]	USA	Quantitative (2004 CAITUS survey data)	1618	Asian Indians In California	NR	Middle	> 18	NR
16	Petti et al. 2018 [25]	Italy	Quantitative Interviews using questionnaire	211	South Asians (Indian = 17.5%, Pakistani = 40.3%, Bangladeshi = 26.1%, Sri Lankan = 16.1%)	M = 100 F = 0	NR	18–73	72

^a^*NR* = Not Reported

**Table 2:** Study findings and quality rating

S.N.	Author Year of Publication Study design	Findings			Quality Rating (scores in %)
		***Knowledge***	***Attitudes***	***Practices***	
1	Summers et al. 1994 [37] Cross sectional study	• 62% perceived pan chewing practice as good, 20% as bad, 13% ‘neither good nor bad’ and 5% ‘did not know” • Participants frequently unaware of their oral condition as well as the harmful effects of Pan	• 4% stated that pan chewing was just a habit and 22% claimed that it was pleasant and refreshing. • 12% claimed that it was good for teeth and gums. 11% thought it “aided digestion” and 6% considered that it relieved pain and had an anti-inflammatory effect. • Believed that it made lips attractive (red) • Pan used in Social gatherings, auspicious occasions and etiquette.	• > 16 quid daily use among heavy pan chewers • Tobacco was employed in smoking, pan chewing and for oral hygiene purposes • 59% women claimed that they spat pan out after finishing chewing pan, 24% swallowed it, 17% stored it in buccal sulcus and 3% were in habit of sleeping with quid in their mouth. • The mean age of onset 17 years, but 51% were started at age of 10 years. • 58% never had dental visits.	B (62.5)
2	Pearson et al. 1999 [38] Cross sectional study	• 43% of participants did not know that pan chewing habit could be bad for health. • More females (49%) than males (38%) were unaware of the harmful effects of pan chewing.	• 23% believed pan chewing habit was good for the health- relieves pain, aids in digestion, freshens mouth and keeps teeth strong. • Females were less likely than males to feel that regular check-ups are important. • Barriers to use of dental services included language, cost and fear (21%) • 64% indicated preference for GP over dentist regarding check-up of mouth ulcer. 39% expressed the wish to learn more about oral healthcare.	• 78% reported habit of pan chewing and half of them developed it by the age of 17. • 14% reported addiction to pan chewing habit. • 33% were tobacco smokers and 64% of them started this habit before the age of 21. • 71% of smokers also chewed pan. • 25% never visited a dentist.	B (75)
3	Shetty et al. 1999 [27] Cross sectional study	• 42% of respondents could not identify early sign of Oral Cancer. • > 50% were not aware of sites of mouth prone for Oral Cancer. • 80% indicated smoking as a possible risk factor for oral cancer. • Misconceptions about the causes of oral cancer such as use of oral contraceptives, removal of teeth and eating sugary food.		• Significant difference seen in Betel quid chewing habit among age groups (42.2% of adults in 50–80-year age group practicing this habit as compared to only 5.3%in 16–29-year age group. • Traditional method of betel quid chewing is being replaced with readily processed areca nut and tobacco products.	B (62.5)
4	Khan et al. 2000 [39] Cross sectional study			• Tobacco chewing habit was found common amongst Bangladeshis (approx. 50%), Indians (> 40%), and Pakistanis (> 20%). • Only 3% of Bangladeshis and Pakistanis reported habit of drinking alcohol while > 20% of Indians were engaged in this habit. • Indians educated beyond the age of 16 years were more likely to chew products containing tobacco. • Less educated Bangladeshis were more engaged in practice of chewing tobacco. • Smoking habits were found less common in Indians (< 10%) and Pakistanis (< 10%) as compared to Bangladeshis (approx.20%).	B (50)
5	Vora et al. 2000 [28] Cross sectional study	• 78% of Sikh males did not know about oral cancer • 10% recognized alcohol as a risk factor for oral cancer • Major sources of knowledge included school/college education, the press and media, and health education leaflets		• The chewing of pan is prevalent among 2nd generation Hindus, Muslims and Jains but low usage was observed among Sikhs. • Sikh males tend to drink alcohol more, whereas Muslim males use tobacco and chew pan	B (62.5)
6	Prabhu et al. 2001 [26] Cross sectional study	• Only few knew about association of pan chewing and oral cancer. • Majority of teenagers have not identified with this cultural norm even if their parents were regular betel quid chewers.	• Many from lower socio-economic status and less inclined to think that it could cause cancer. • More likely to agree that pan tasted good. • Tended to think it made their teeth and gums stronger	• Median age of first chewing - 9 years • Similar proportions of adolescent males and females chewed pan • 28% chewed Pan & 51% of whom chewed on most days	B (62.5)
7	Changrani et al. 2006 [21] Piot study	• Bangladeshis more likely to identify pan as a cause of oral cancer than Indians (66% vs 48%) • Indians identified gutka as a cause of oral cancer more correctly than Bangladeshi (93% vs 60%)	• Health benefits of pan were cited as “relieves constipation,” “improves stamina,” “fights cold,” relieving tension, and for mood improvement. • Pan also believed to cause harms like cancer, dental problems, ulcers, addiction, and hypertension	• The communities migrated with pan and gutka use habits • Pan was popular in Bangladeshis while gutkha use considerably limited.	B (50)
8	Croucher et al. 2011 [40] Cross sectional study	• Superior oral cancer knowledge following campaign awareness. • Younger male respondents with some completed education more likely to be aware of oral cancer		• Limited dental attendance as compared to medical visits	B (75)
9	Siddique et al. 2013 [41] Pre and Post intervention study	Gutka was the most correctly identified risk factor among first generation females (50%) and second-generation males and females (63 and 69% respectively).		• First generation Gujarati Muslim males had the highest proportion of regular supari users (33%), greater than their female counterparts (12%) • Complete absence of regular gutka use in Gujarati Muslims except among first generation males (42%)	B (62.5)
10	Lokhande et al. 2013 [22] Grounded theory case study	• Mixed understanding about harmful effects of chewing tobacco. • More knowledge about ill effects of smoking.	• Flavoured gutka for “fresh breath” • Find chewing mentally stimulating, gives pleasure, improved their mood and helped them relax • Get the supply from friends of India or Fiji due to ban in New Zealand • Cultural norms as barrier to cease tobacco chewing	• Gutka was preferred choice for chewing tobacco. • Use ranged from twice a day to 12 times a day • Daily use ranged from twice a day to 12 times a day	A (88.8)
11	Banerjee et al. 2014 [23] Focus group study	• Acknowledgment of addiction • Scepticism about the pan-cancer link	• Compensatory beliefs • SATP believed to relieve boredom, aid in digestion after meals, reduce stress, and to increase alertness • Encouraged by pleasant sensations of smell, taste and cosmetic benefits	• Early age initiation • Easy availability • Habit inherited from generations • Changed patterns of gutka/tambaku pan use behaviour after immigration	A (88.8)
12	Merchant et al. 2016 [24] Cross sectional study	• Pakistani and Bangladeshi more likely to have low knowledge as compared to Indians. • Followers of Islam were found low knowledge than Hindus. • Males, and the better educated, more likely to report risk factors for oral cancer		• 42% of total subjects used tobacco, Gutka or Pan in combination with alcohol; while 41% people stated habit of Smoking and 5% reported tobacco chewing habit. • Participants of Indian or Sri Lankan ethnic origin were more likely to consume alcohol than those of Pakistani, Bangladeshi or Malaysian-Indian origin. • Rare dental visits reported	B (50)
13	Hrywna et al. 2016 [42] Focus group study	• Variety of opinions about the classification of SATP • Awareness about health risks regarding use of tobacco products	• Use of SATP common at social gatherings or after meals. • Perceived benefits with use of SATP like stress relief, relaxation, relieving boredom, mouth cleanse and as an aid for digestion.	• > 70% reported having tried at least one SATP and more than half (51.5%) currently use a SATP. • Native born older males described gutkha as the most common SATP while native born older females described pan/pan masala as the most popular products	B (77.7)
14	Shi et al. 2017 [43] Pre and Post intervention study	• 52.3% believed AN alone could cause cancer • Overall low understanding of AN’s carcinogenic properties	• Perceived harms like addiction, kidney stones and thinning of blood	• 64.6% used AN • 8.2% reported social use • 28.6% reported usage during celebrations only, and 28.6% reported daily use.	C (37.5)
15	Mukherjea et al. 2018 [44] Based on old CAITUS cross sectional study			• Integral religious practices with CST use • The prevalence of current CST use was 13.0% (14.0% for men and 11.8% for women). • More CST use was reported by AIs who had a college degree or higher level of education, were born in India, and were practicing Hinduism.	C (37.5)
16	Petti et al. 2018 [25] Cross sectional study	• knowledge about oral carcinogenicity of BQ was lower among chewers (41.2% vs 46.6%). • Lack of awareness toward oral cancer and other BQ chewing-related diseases.	• Significantly associated attitudes were being a routine smoker, being born to parents who were also chewers, the perception that chewing is good for health (43.6%) and that it helps to relieve stress. • two- thirds believed that pan chewing helps to relieve stress, while 17% stated that it led to stress relief	• The high BQ chewing prevalence rate (40%) in immigrants from the Asia / Indian subcontinent reported • BQ usage, along with smoking and tobacco chewing, as an integral part of the lifestyle of these people before and after migration	B (75)

*SATP* South Asian Tobacco products, *AN* Areca Nut, *BQ* Betel Quid, *AI* Asian Indians, *CST* Cultural Smokeless Tobacco

A = all or most of the criteria have been fulfilled (a score of 80–100%); B = some of the criteria have been fulfilled (50–79%); and C = few or none of the criteria have been fulfilled (< 50%)

### Definition of terms

For the purpose of this review, high-income countries with developed economies such as the United States of America, the United Kingdom, Canada, Australia, New Zealand have been referred to as ‘developed countries’ [45]. The terms ‘knowledge’, ‘attitudes’ and ‘practices’ have been used widely in this paper. The ‘Knowledge’ is the capacity to acquire, retain and use information; a mixture of comprehension, experience, discernment, and skill [46]. The ‘Attitudes’ refer to inclinations to react in a certain way to certain situations; to see and interpret events according to certain situations; to see and interpret events according to certain predispositions, or to organize opinions into coherent and interrelated structures [46]. The ‘Practices’ is the application of rules and knowledge that leads to action [46]. For the purpose of this paper; the terms of knowledge, attitudes and practices have been refined in relation to oral cancer risk. The term ‘knowledge’ in this paper refers to one’s awareness, level of information and understanding regarding the oral cancer risk. The term ‘attitudes’ has been used here to depict the inclinations, perceptions, and beliefs of the people associated with oral cancer risk. The term ‘practices’ here relates to a person’s oral cancer risk related habits and the actions regarding initiation, continuation or quitting of these habits.

## Results

### Study selection summary

The search of databases identified 162 records; 41 were duplicates and subsequently removed. A further 7 articles were found through a manual search of reference lists of identified studies which resulted in a total of 128 articles. The process of initial screening based on title and abstract resulted in the exclusion of 94 articles, leaving 34 for full-text screening. After full-text review, a further 18 articles were excluded as they were literature reviews (*n* = 5) and a case report (*n* = 1), did not focus specifically on oral cancer-related knowledge, attitudes and practices (*n* = 10), and were conducted in upper middle income countries (*n* = 2) (See Additional file 5 for Table of excluded studies). This resulted in 16 studies for inclusion in this review; three were qualitative [20, 22, 42] and 13 were quantitative [21, 24–28, 37–41, 43, 44]. (See Fig. 1 for the study selection process).

### Study characteristics

The 16 studies included in this review were published between 1994 and 2018 and were conducted across four countries namely, United Kingdom (UK; *n* = 9), United States of America (USA; *n* = 5), Italy (*n* = 1), New Zealand (NZ; *n* = 1). Table 1 shows the salient features of the studies included in this review. The sample size (see Table 1 for study characteristics) of the studies ranged from 10 to 1618 participants with a total of 4772 in number. Participants were immigrants mainly from India, Pakistan and Bangladesh and consisted of first to third generations. The age of the participants ranged from 12 to 87 years and consisted of mostly males [20–22, 24–28, 38, 43]. Nine of the studies addressed all the themes of the oral cancer risk-related knowledge, attitudes and practices among South Asians in developed countries [21, 24–28, 37, 38, 43]. One quantitative study [25] mentioned use of validated questionnaire while five other quantitative studies [26, 27, 37–39] reported use of previously pilot-tested survey.

### Quality of the included studies

The quality of the studies was rated as good (*n* = 2) (score ≥ 80), fair (*n* = 12) (score 50–79%) and poor (*n* = 2) (score < 50%) (see Table 2 for study findings and quality rating). Due to limited available literature in this area, all the studies were included in this review irrespective of their quality, to allow the reader to make their own judgement.

(see Additional file 6 for critical appraisal of articles)

### Study findings

The findings of this review were categorised under themes of Oral cancer knowledge, Oral cancer attitudes and Oral cancer practices which are explained below:

### Theme 1: Oral cancer knowledge

Fourteen studies [20–22, 24–28, 37, 38, 40–43] explored the knowledge of South Asian immigrants regarding the oral cancer risk. These studies assessed the level of information as well as awareness of the participants in relation to the risk of oral cancer associated with the consumption of alcohol, tobacco and areca nut preparations. Most of the studies reported a general lack of knowledge (43–76%) regarding oral cancer risk across respondents from South Asian subgroups irrespective of the native country, age, gender and social class [21, 25–28, 37, 38, 40]. Few studies though did find an association between knowledge levels and religion/ethnicity. Pakistanis (69%) and Bangladeshis (85%) were reported having ‘low knowledge’ of oral cancer risk when compared to those of Indian (47%) ethnicity [24]. However, Bangladeshi immigrants (66%) were found more likely to identify ‘pan’ as a possible cause of oral cancer than Indian-Gujarati (48%) immigrants in the USA [21]. The adequate knowledge regarding oral cancer risk was also associated with religion, as Sikh participants were found less aware of oral cancer risk factors when compared to Muslim and Hindu participants [24, 28].

According to Shetty et al. there were many misconceptions among participants regarding possible causes of oral cancer including the use of oral contraceptives, removal of teeth and eating sugary food [27]. In contrast, a few studies did show that participants had knowledge (58–69%) about one or more risk factors responsible for causing oral cancer like smoking, alcohol use and gutka chewing [24, 41, 43]. This information was more common among more educated and second-generation individuals especially males [24, 38, 41, 43]. Sources of knowledge among participants included school/college education, press or media, relatives (27–43%), health education leaflets/awareness campaigns (24–57%), dentists (16–33%) [28, 40, 41].

Four studies also showed that even if respondents were aware of the harmful effects of chewing tobacco and alcohol use, there was scepticism regarding the association of pan/gutka with oral cancer [20, 22, 25, 42]. Similar qualitative findings were reported by Lokhande et al. [22], Hrywna et al. [42] and Banerjee et al. [20] as they found mixed understandings prevalent among participants regarding oral cancer risk:

“*There is a mixture of happiness and sadness, but I sometimes feel sad and very low.*. *. I think there is “100% health risk” to chew tobacco which can cause mouth disease*.”(page 48) [22].

*“I think supari is the most popular, that’s not on the [survey] …*. *When I was younger I never even knew it was tobacco … I might have even put one in my mouth because I didn’t know. It didn’t even taste that bad from my memory. I would say supari and gutkha.”* (page 5) [42].

### Theme 2: Oral cancer attitudes

The attitudes of South Asian immigrants towards oral cancer risk were reported in nine studies [20–22, 24–28, 37, 38, 40–44]. The relevant attitude items mainly were related to beliefs regarding the association of risk products with oral cancer, perceived benefits as well as harms of oral cancer risk practices and the context of the use of these risk substances. Some of the studies highlighted that the overall attitude of participants towards oral cancer risk was negative and unfavourable [25, 26, 37]. Poor beliefs were reported among participants (17–41%) regarding preventive health behaviours and modification of risk practices [24, 26, 27, 37, 38]. One study in UK involving Bangladeshi migrants found females were less likely than males to regard regular dental check-ups as important for a healthy mouth [38].

Four studies [25, 26, 37, 38] found that people perceived betel quid/pan/gutka chewing habit good for their health (12–43.6%) which makes ‘teeth and gum stronger’ and believed that it helps them to reduce stress (11.6–51%), relieve boredom with refreshing feeling (22–44%). These findings were reiterated by participants in the qualitative studies by Hrywna et al. [42] and Banerjee et al. [20]:

*“It has benefit; it can be therapeutic too sometimes,”* (page 7) [42].

*“And there are people who feel good; they think it releases tension/worries. So sometimes I think that having a little can cool your mood if you are feeling angry or annoyed.”* (page 535) [20].

Other specific health benefits of betel quid/pan/gutka perceived by participants included aiding in digestion (11–33.6%) and pain relief (6–34.1%) [21, 25, 26, 37, 38]. Furthermore, some studies found that use of pan/gutka was also encouraged among South Asians due to its fragrant smell (12.6%) [26], pleasant taste (35–37.4%) [25, 26, 37, 43] and cosmetically appealing red staining on lips [26, 37]. Some people were found consuming areca nut preparations just out of habit and for refreshment (3.3 to 42.7%) [25, 26, 37, 43]. Furthermore, such risk habits were found more popular among people from lower socio-economic status, who were less inclined to think about oral cancer risk associated with these products [21, 25, 26, 37].

Similar views were highlighted in the qualitative studies [20, 42]:

*“I find the smell of it very pleasant when I chew it. When someone else eats, I am attracted to the smell. That’s why I eat it*.” (page 535) [20].

*“To feel good or get a buzz. I’m sure that’s why people use it.”* (page 7) [42].

Respondents perceived few harms associated with areca nut products like dental problems, chest pain, hypertension and kidney stones [27, 43].

Some studies revealed wide cultural acceptability of areca nut products during festivals celebrations and special occasions (7.1–18.2%) [24, 25, 43]. The use of tobacco-related products such as hookah, pan, and supari were found common at social gatherings or after meals [42, 43]. Moreover, people believed that society played an important role in influencing their habits [20, 22, 42] and it was hard to refuse offers of these products [22]:

“*My friends chew it and I cannot say no to them when they offer – it is rude to say no in our culture.*. *. Every third person in Pakistan chews tobacco.”* (page 48) [22].

*“I think paan is always a tradition at parties and weddings. A lot of these chewing things like supari and gutkha, I’ve seen when I was in India … the older men, after they eat their food or if they’re going on a walk they just pack a lip …*.” (page 6) [42].

One study in the USA found the use of tobacco and areca nut preparations among older South Asians helped them connect to their homeland [42].

“...*If you go to Jersey City or Iselin [cities in New Jersey with large South Asian populations], you’ll see it’s something that’s so deeply rooted in their culture that it’s ok for us to do it. It justifies everything*”. (page 7) [42].

### Theme 3: Oral cancer practices

All studies [20–22, 24–28, 37–43, 47] explored the aspects of oral cancer risk related practices and reasons behind the initiation of these habits among South Asian immigrants. Up to 50% of participants were found engaged in one or more negative oral cancer risk related practices like smoking, alcohol drinking, chewing of betel quid and tobacco [20, 22, 24–28, 37–39, 44]. Pan/Betel quid chewing was revealed as the most popular practice (40–97%) followed by smoking and gutka chewing [25, 26, 37, 38]. Followers of Islam (8–23%) were found less likely to consume alcohol when compared to Sikh (43–100%) and Hindu communities (27.6–64%) [24, 28], Whereas, areca nut and pan use were found more common among Muslim participants (24–69%) along with Hindu (32–71%) and Sikh participants (0–95%) [24, 28, 41]. A study in UK involving a number of ethnic groups found that Indians educated beyond the age of 16 years were more likely to chew tobacco products while in the Bangladeshi population the contrary was true [39].

There were also notable age variations when the risk habits were initiated in their home countries ranging from 3 to 18 years [20, 21, 26, 37, 38]. Various reasons were cited behind the initiation of these practices such as social networks made up of South Asian friends or co-workers (45–48.2%), passing of habit from one generation to the next (3.3–81%), observation and encouragement within family members (27.5–81%) [21, 25, 26, 43, 44]. These findings were also reflected in the qualitative studies [20, 22, 42] as indicated in the quote below:

“*From observing. Mother would have it. Grandmother would have it. Aunts use it. When everyone would have it, I would have it too. To see what it’s like.*” (page 535) [20].

*“I must have influenced my son to get addicted to chew tobacco.”* (page 48) [22].

Despite legal restrictions in developed countries, the easy availability of gutka/customisable pan in Asian grocery stores, restaurants, specialised pan stalls, and supermarkets was highlighted as a factor responsible for the continuation of risk practices among respondents [20, 22, 43]. Similar views were raised in focus groups by Banerjee et al. [20]:

“*One of my brothers here said that it can be found in Pakistani...I mean Indian and Bangladeshi stores. Other stores don’t sell it, it’s true. Meaning...it is used by Bangladeshi and Indians as well...If some- one says it is restricted, I won’t agree. Not so much*.” (page 534) [20].

A pilot study [21] in the USA revealed that immigration can also influence the patterns of risk practices with participants switching habits from pan chewing to gutka use (nearly 54%) due to the social unacceptability of the former and ease of procurement /storage of the latter. Supporting this notion is a study in the USA that found that people preferred smoking and sometimes swallowing the tobacco/pan instead of spitting it out because of society finding this inappropriate [20]. However, some studies found that betel quid usage along with tobacco chewing/smoking was an integral part of lifestyles, deeply rooted in the culture of south Asians and that these practices simply continued in new settlements as a habit or addiction [25, 26, 42–44].

Studies also explored different actions and perspectives of South Asian immigrants on quitting oral cancer risk-related practices and found a general interest among respondents (30–80%) in quitting their risk practices [25, 26, 28, 37, 43]. However, quitting these practices was acknowledged to be difficult among users (18.2–38%) [25, 26, 28, 43] who attempted to quit. Participants highlighted the role of self-motivation [20, 22], doctor/dentist [20, 24, 27, 37, 41, 43] as well as government checks [20, 22] in curtailing their use of tobacco/pan products. However, participants did not regularly see a dentist (4–58%) but gave priority to visit general medical practitioners (39–91.3%) especially in case of medical need [24, 27, 37, 38]. Furthermore, general practitioners were found to usually lack knowledge about gutkha/pan use among South Asians [20, 43] and hence rarely discussed the ill-effects of these products during the consultation [20, 27, 37, 43]. Similar findings were reported by Banerjee et al. [20] in their qualitative study:

*“Now that we go to the doctor, doctor asks do you smoke, do you drink. That’s all, not more than that. But they don’t say that you should not touch this at all. They don’t say that.*” (page 537) [20].

## Discussion

This is the first integrative review to assess current evidence regarding the knowledge, attitudes, and practices of South Asian immigrants in relation to oral cancer risk in developed countries. The majority of studies were conducted in the USA [20, 21, 42–44] and UK [24, 26–28, 37–41], and more recently in Italy [14] reflecting the changing migratory patterns of South Asians. It is also evident from the diversity of populations studied that irrespective of native countries, the oral cancer risk behaviours are widespread across a broader age range, gender, generations, and social class.

Overall, this review shows a general lack of oral cancer risk-related knowledge among South Asian immigrants in developed countries with persistent low levels of information [21, 25–28, 37, 38, 40]. The scepticism and confusion regarding the link of areca nut/betel quid with oral cancer existed even among the well- informed South Asians [20, 22, 25, 42]. This finding echoes the observation from a study conducted in a developing country (South Africa), where more than half of the South Asians were unaware of health risks associated with the areca nut chewing [48]. It is also consistent with a systematic review exploring the social context of smokeless tobacco use in the South Asian population which found low levels of knowledge in this population regarding harmful health effects associated with the use of smokeless tobacco [49]. These similarities in findings suggest that South Asian immigrants have limited knowledge about oral cancer risk products regardless of their country of settlement. Similar to a recent research around areca nut chewing in Sri Lankan adolescents [50], the study findings showed that more educated migrants, particularly second-generation males were more likely to present better knowledge and level of awareness around risk products linked to oral cancer [24, 41, 43]. Surprisingly, school and university education were identified by participants as the primary source of knowledge in this area rather than awareness campaigns and advice received from health professionals including dentists [28, 40, 41]. These results reiterate Mukherjea et al.’s [51] call for a universally standard and consistent classification of smokeless carcinogenic products as tobacco products among clinicians, researchers, and policymakers to improve knowledge and awareness among South Asian people. This also supports the suggestion by Awan et al. for employment of well-structured programmes for South Asians in terms of educating them about the health hazards of smokeless tobacco [52].

The level of knowledge around oral cancer risk factors among South Asians seems to be influenced by ethnicity and religion to some extent. The findings suggest that the South Asian community should not be classified as a homogenous group when formulating preventative strategies, because as also noted by Williams et al. [53, 54], South Asian population subgroups from different ethnic origins and varied religions present differences in risk factors, level of knowledge as well as health-related behaviours. This review indicates that a clear understanding and better assessment of the concepts regarding religion and ethnicity will help improve specific oral cancer risk awareness strategies among South Asian subgroups. Interestingly though none of the studies explored the impact of socioeconomic status on oral cancer related knowledge and awareness. This is an area that should be explored further in future studies particularly as this connection has been well documented in other areas [55–57].

The rigid beliefs of South Asian immigrants regarding the use of tobacco and areca nut products may be contributing to their negative attitudes towards oral cancer risks. This review revealed the poor beliefs and ignorant perspective of South Asians towards preventive health behaviours and modification of risk practices [24, 26, 27, 37, 38, 42]. Despite associated oral cancer risks, the perceived benefits of these products influenced many South Asians particularly those from lower socio-economic status [21, 25, 26, 37], to continue using risk products like betel nut/quid, gutka even after immigration. These results are further validated by another systematic review conducted around the use of smokeless tobacco in South Asians, which found respondents had more perceived health benefits than ill effects from using these risk product [49]. These findings strongly highlight an un-informed viewpoint of South Asian immigrants towards oral cancer risk which needs to be further explored, to deliver a more targeted and specific educational approach. Prabhu et al. [26] advocate the need for a Common Risk/Health Factor Approach (CRHFA) to improve awareness regarding particular ill effects related to any risk product rather than orienting it to oral cancer alone.

This review also explored the cultural perspective behind the use of oral cancer risk products among South Asians. The use of tobacco and areca nut preparations was found to be widely acceptable as cultural tradition during special occasions/festivals [24, 25, 43] which is further influenced by socialisation [20, 22, 42] and connection to their homeland [42]. These findings are consistent with a review by Mukherjea at al [47], which highlighted culturally-specific use of tobacco products among South Asian immigrants and suggested the need for a more detailed assessment on the use of such products. Since educational interventions and awareness campaigns in relation to oral cancer [40, 41] have proven effective in the past to improve the level of information among south Asian immigrants, community-based and culturally-tailored efforts are needed to change the social norms associated with the use of such risk products.

Lastly, a notable finding was that up to half of the respondents engaged in the risk practices such as smoking and chewing tobacco, areca nut products [20, 22, 24–28, 37–39, 44]. These practices were popular across almost all age groups and generations [20, 21, 26, 37] with various patterns of practices in different religions [24, 28, 41]. Of concern was the supportive role of family and friends in the initiation of this kind of practices [21, 25, 26, 43, 44]. These findings complement the recent WHO report [58] regarding trends of tobacco product use in the South-East Asia region. This review also echoes the higher frequency of these risk practices among South Asian immigrants in developed nations as reported by Health Survey of England 2004 [59] and CAITUS (California Asian Indian tobacco use survey) of California 2004 [60]. Easy availability of tobacco and areca nut product despite legal restrictions [20, 22, 43] was explored as an important factor in the continuation of risk practices among South Asians after immigration as well. This is in line with Awan et al. who observed higher consumption rates of such risk products due to cheap prices, easy accessibility and heavy marketing [61] in the native countries of South Asians. This review suggests the need for strengthening of government efforts and legislation around sale as well as health warning requirements specifically for smokeless tobacco products in developed countries.

Migration also had an effect on the usage of risk products [20, 21] among South Asians sometimes leading to people switching from one habit to another due to social unacceptance. Unfortunately, the success rates for quitting these practices were disappointingly low among the South Asian population despite some understanding of health risks associated with risk habits [25, 26, 28, 37, 43]. This reiterates the findings from study conducted in Malaysia, where majority of Indian immigrants perceived the habit of smoking and alcohol consumption difficult to give up [62]. Since quitting of these risk habits was difficult for participants, the need for the government and health care providers to play a more active role in this area was advocated in a number of studies [20, 27, 37, 43]. These findings highlight the need for more effective intervention strategies to address the oral cancer risk-related practices among South Asian immigrants. These findings also support the recommendations by Mukherjea et al. [47] for different approaches at the individual, community, organizational and policy levels to curtail the use of tobacco products. The role of media [47] to change socio-cultural norms among South Asians and appropriate counselling at medical/dental centres to support quitting these practices should also be advocated.

### Implications of the findings

The study findings have significant implications for the development and implementation of preventative interventions to address oral cancer risk practices among South Asian immigrants. Considering the high prevalence of oral cancer in South Asian countries, the development of effective culturally sensitive programs is necessary to increase awareness among at-risk populations in developed countries. Appropriate screening and counselling regarding use of risk products should be provided through general practices as well as dentists. Community organisations should be involved in promoting the cessation of tobacco areca nut preparations at cultural events and festivals. The role of media/social media advertising and more targeted educational campaigns should also be explored to raise understanding among people about good oral health behaviours while minimising oral health risk habits. In addition, policy makers need to strengthen existing legislation regarding the sale of tobacco, areca nut products and the development of accessible oral cancer awareness resources. These findings also have implications for future research particularly in countries that currently have an active migration program and are attracting South Asian immigrants like Canada and Australia. It is important that further research is undertaken in these countries to confirm whether the review findings are relevant and inform preventative strategies in this area.

### Limitations

The studies included in this review varied in methodology as well as quality and hence, the reliability of these studies may be compromised. There is also a lack of information regarding the validated questionnaires and confounding factors in most of the studies which may have affected the results. The South Asian population is broad and findings from some studies may not be generalisable to all South Asians. This review has not included articles that were unpublished or published in other languages and therefore, all studies in this area may have not been retrieved. Moreover, comparisons between studies were too difficult given different methods employed and thus, this review has placed little focus on such comparisons considering these variations, but rather has tried to illustrate an overall picture. All these limitations should be taken into account for designing future studies to ensure reproducible and generalisable evidence.

## Conclusion

This integrative review confirms that South Asian immigrants in developed countries have inadequate oral cancer risk-related knowledge, poor attitudes towards oral cancer risk and a strong inclination towards negative oral cancer risk practices. From this review, it appears that they are ill-informed regarding health risks associated with the use of risk products especially tobacco, areca nut products and are also not receiving appropriate information in this area. The unpredictable and constantly changing migration pattern of South Asians are also concerning in the current scenario. In light of these facts, a multidisciplinary approach involving health professionals, community organisations and policymakers is required to promote oral cancer awareness among this population. Further, designing culturally relevant preventative strategies and educational programs is needed to encourage cessation of risk habits among South Asians.

## Supplementary information

**Additional file 1.** Search strategy/terms.

**Additional file 2.** Full text screening of articles.

**Additional file 3.** CASP checklist.

**Additional file 4.** JBI checklist.

**Additional file 5.** Table of excluded studies.

**Additional file 6.** Critical appraisal of articles.

## Data Availability

Data sharing is not applicable to this article as no datasets were generated or analysed during the current study.

## Abbreviations

SATP: South Asian Tobacco products
AN: Areca Nut
BQ: Betel Quid
AI: Asian Indians
CST: Cultural Smokeless Tobacco
