# Supplementary material for: Knowledge, attitudes and practices of South Asian immigrants in developed countries regarding oral cancer: an integrative review
Source: BMC Cancer. 2020 May 27;20:477. doi: 10.1186/s12885-020-06944-9 (PMC7251750; doi:10.1186/s12885-020-06944-9)
Supplement: Supplementary file 1 — Additional file 1. Search strategy/terms. [file 12885_2020_6944_MOESM1_ESM.pdf]

Database: Ovid MEDLINE(R) ALL
Search Strategy:
----
1 Mouth Neoplasms.mp. or Mouth Neoplasms/ (34703)
2 "oral cancer".mp. (12360)
3 ("oral cancer" or "mouth cancer" or "mouth neoplasms" or "oral tumours").mp. [mp=title, abstract, original title, name of substance word, subject heading word, floating sub-heading word, keyword heading word, organism supplementary concept word, protocol supplementary concept word, rare disease supplementary concept word, unique identifier, synonyms] (38760)
4 Asians.mp. (15275)
5 "south asian".mp. (4978)
6 Immigrants.mp. or "Emigrants and Immigrants"/ (23679)
7 ("India" or "Pakistan" or "Sri Lanka" or "Nepal" or "Bangladesh").mp. [mp=title, abstract, original title, name of substance word, subject heading word, floating sub-heading word, keyword heading word, organism supplementary concept word, protocol supplementary concept word, rare disease supplementary concept word, unique identifier, synonyms] (195391)
8 Oropharyngeal cancer.mp. or Oropharyngeal Neoplasms/ (6840)
9 Mouth Neoplasms/ or Oropharyngeal Neoplasms/ or Oropharyngeal tumours.mp. (38832)
10 1 or 2 or 3 or 8 or 9 (43864)
11 4 or 5 or 7 (212154)
12 6 and 11 (1568)
13 10 and 12 (15)
