# Supplementary material for: Knowledge, attitudes and practices of South Asian immigrants in developed countries regarding oral cancer: an integrative review
Source: BMC Cancer. 2020 May 27;20:477. doi: 10.1186/s12885-020-06944-9 (PMC7251750; doi:10.1186/s12885-020-06944-9)
Supplement: Supplementary file 2 — Additional file 2. Full text screening of articles. [file 12885_2020_6944_MOESM2_ESM.docx]

| S.No. | Article topic | Authors | Decision | Explanation | Database/researcher |
| --- | --- | --- | --- | --- | --- |
|  | Chewing tobacco use among South-East Asian men in Auckland | Lokhande, Sunder  Glover, Marewa  Selket, Kyro  2013 | Included | explored attitudes, perceptions and practices of chewing tobacco by South Asian men in Auckland (included semi structured interviews with 10 people)  Qualitative | CINAHL/NS |
|  | An assessment of oral cancer curricula in dental hygiene programmes: implications for cancer control | Thacker, K. K.  Kaste, L. M.  Homsi, K. D.  LeHew, C. W.  2016 | Excluded | Survey among faculty members of associate degree Dental hygiene programmes in Illinois, USA (included questions related to oral cancer training components of their programme) | CINAHL/ NS |
|  | Paan and Gutka Use in the United States: A Pilot Study in Bangladeshi and Indian-Gujarati Immigrants in New York City | Changrani, J.  Gany, F. M.  Cruz, G.  Kerr, R.  Katz, R.  2006 | Included | The study was the exploration of practice, attitudes /beliefs and perceptions regarding paan and gutka usage by south Asians (Bangladeshi and Indian-Gujarati immigrant) in New York.  Quantitative (108 item questionnaire & 138 participants) | OVID MEDLINE/ NS |
|  | Areca nut use following migration and its consequences | Warnakulasuriya, S.  2002 | Excluded | Review on available data on the prevalence of areca chewing migrant populations. | OVID MEDLINE/ NS |
|  | Betel quid chewing among adult male immigrants from the Indian subcontinent to Italy | Petti, S.  Warnakulasuriya, S.  2018 | Included | Survey was conducted aiming at investigation of Betel quid chewing prevalence, perceptions and attitudes among immigrants from the India, Sri Lanka & Bangladesh in Rome, Italy.  Quantitative (survey of 200 people) | EMBASE/ NS |
|  | Paan and Gutka in the United States: An Emerging Threat | Changrani, Jyotsna  Gany, Francesca  2005 | Excluded | Overview of Pan and gutka chewing among south Asian immigrants in USA and associated rise in oral cancer cases. (pre-read article in series of next original article by same author involving pilot study) | EMBASE/ NS |
|  | Assessing the oral cancer risk of South-Asian immigrants in New York City | Ahluwalia, K. P.  2005 | Excluded | Reference given about cross sectional study to be conducted to assess oral cancer related knowledge, opinions and practices of 150 south Asian adults (>60 yrs) in New York. | SCOPUS/ NS |
|  | Areca nut and betel quid chewing among South Asian immigrants to Western countries and its implications for oral cancer screening | Auluck, A.  Hislop, G.  Poh, C.  Zhang, L.  Rosin, M. P  2009 | Excluded | Overview and discussion of the sociocultural beliefs, knowledge and practices regarding betel quid/areca nut chewing and discuss its implications for oral cancer screening among south Asian immigrant population. | SCOPUS/ NS |
|  | Areca nut and tobacco chewing habits in Durban, KwaZulu Natal | Bissessur, S.  Naidoo, S.  2009 | Excluded | Cross sectional study conducted South Africa (upper middle-income country) to investigate the prevalence of areca nut chewing, associated habits and awareness of risk factors among migrant communities from India, Pakistan, and Dubai. | SCOPUS /NS |
|  | Oral cancer in Australia--risk factors and disease distribution | Cox, S.  2000 | Excluded | Overview of incidence of oral cancer and discussion on related practices and knowledge of south Asian immigrant communities. | SCOPUS/ NS |
|  | Betel quid chewing and the risk of oral and oropharyngeal cancers: A meta-analysis with implications for cancer control | Guha, N.  Warnakulasuriya, S.  Vlaanderen, J.  Straif, K.  2014 | Excluded | Meta-analysis of 50 publications assessing the relationship between oral/oropharyngeal cancer and chewing betel quid among Asians (nothing about K/P/A of south Asian immigrants related to oral cancer risk) | SCOPUS/ NS |
|  | Oral submucous fibrosis: Two cases of malignant transformation in asian immigrants to the United Kingdom | McGurk, M.  Craig, G. T.  1984 | Excluded | OSF cases among Asian immigrants in UK, are described with clinical presentation, etiology and treatment. (nothing about K/P/A of south Asian immigrants related to oral cancer risk) | SCOPUS / NS |
|  | Nasopharyngeal and hypopharyngeal carcinoma risk among immigrants in Sweden | Mousavi, S. M.  Sundquist, J.  Hemminki, K.  2010 | Excluded | High rates of hypo-pharyngeal carcinoma risk in immigrants (specifically Indian) in Sweden mentioned.  Family cancer database used | SCOPUS/ NS |
|  | Moving toward a true depiction of tobacco behavior among Asian Indians in California: Prevalence and factors associated with cultural smokeless tobacco product use | Mukherjea, A.  Modayil, M. V.  Tong, E. K.  2018 | Included | California Asian Indian Tobacco Use Survey data from 2004 (n= 1618) were used to investigate CST prevalence among California’s AIs. CST products included paan, paan masala, and gutka.  Old population survey used to categorize beliefs/attitudes of immigrants regarding oral cancer risk. | SCOPUS/ NS |
|  | 'Betelmania' - Betel quid chewing by Cambodian women in the United States and its potential health effects | Pickwell, S. M.  Schimelpfening, S.  Palinkas, L. A.  1994 | Excluded | Survey undertaken of group of Cambodian refugee women who were addicted to betel nut and its associated components. ( no interview of south Asians) | SCOPUS/ NS |
|  | Oral submucous fibrosis in a 31-year-old Indian woman. First case report from Germany | Reichart, P. A.  Philipsen, H. P.  2006 | Excluded | A case report of Indian woman with OSF in Germany described along with mention of areca nut chewing in South Asia. (article full text not available in English) | SCOPUS/ NS |
|  | Evaluation of a culturally tailored smoking prevention program for Asian American youth | Ma, G. X.  Lan, Yajia  Edwards, R.L.  2004 | Excluded | This study evaluated effectiveness of a smoking prevention program for Asian American youth.  Nothing specific about knowledge, attitudes and practices of South Asian immigrants regarding oral cancer risk | SCOPUS/ NS |
|  | Oral Cancer Awareness among community-dwelling senior citizens in Illinois | Posorski, Ewa  Boyd, Linda  Giblin, Lori  Welch, Lisa  2014 | Excluded | The study assessed oral cancer awareness among senior citizen in Illinois.  Nothing specific about knowledge, attitudes and practices of South Asian immigrants regarding oral cancer risk | SCOPUS/ NS |
|  | Alcohol tobacco and pan use and understanding of oral cancer risks in Asian males in Leicester | Vora, AR  Yeoman, CM  Hayter, JP  2000 | Included | Study to determine use of alcohol, tobacco and paan among males from the various Asian communities in Leicester; and assess their knowledge and attitudes towards oral cancer risk factors and prevention. Also, to determine any differences regarding habits and attitudes between first- and second-generation Asians.  Questionnaire | SCOPUS/ NS |
|  | Knowledge, attitudes and beliefs of adult South Asians living in London regarding risk factors and signs for oral cancer | Shetty, K. V.  Johnson, N. W.  1999 | Included | Study conducted to assess the knowledge, attitudes and beliefs of south Asian adults regarding the risk factors and signs for oral cancer  Questionnaire and structured interviews | SCOPUS/ NS |
|  | Predictors of tobacco and alcohol consumption and their relevance to oral cancer control amongst people from minority ethnic communities in the South Thames health region, England | Khan, F.A.  Robinson, P.G.  Warnakulasuriya, K.  Newton, J.T.  Gelbier, S.  Gibbons, D.E.  2000 | Included | Study conducted to examine the determinants of the health behaviour of ethnic groups in relation to alcohol and tobacco use. Self-classified ethnic groups were identified: Black-African; Black–Caribbean; Indian; Pakistani; Bangladeshi and Chinese/ Vietnamese.  Questionnaire | SCOPUS/ NS |
|  | Dental service use and the implications for oral cancer screening in a sample of Bangladeshi adult medical care users living in Tower Hamlets, UK | Pearson, N.  Croucher, R.  Marcenes, W.  O’Farrell, M.  1999 | Included | Study to assess the use of dental services, barriers to uptake of dental care and attitudes to regular dental examinations and the prevalence of tobacco and paan chewing habits in a group of Bangladeshi medical care users.  Questionnaire | SCOPUS/ NS |
|  | Campaign awareness and oral cancer knowledge in UK resident adult Bangladeshi: a cross-sectional study | Croucher, R.  Islam, S. S.  Nunn, H.  2011 | Included | Reports awareness of the ‘Open up to Mouth Cancer’ campaign materials and oral cancer knowledge among UK adult Bangladeshi communities, which are at high risk for oral cancer.  Quantitative (survey of 340 immigrants) | PROQUEST CENTRAL/ NS |
|  | Ethnicity and oral cancer | Scully, Crispian  Bedi, Raman  2000 | Excluded | Review article: Consideration of evidence of marked ethnic variations in the incidence, management, and survival of oral cancer, and then, to review possible explanations for these variations in Intercountry context. | PROQUEST CENTRAL/ NS |
|  | Association of Betel Nut with Carcinogenesis: Revisit with a Clinical Perspective | Sharan, Rajeshwar N.  Mehrotra, Ravi  Choudhury, Yashmin  Asotra, Kamlesh  2012 | Excluded | Systematic review attempts to put in perspective the consequences of this widespread habit of BN/BQ mastication, practiced by approximately 10% of the world population, on oral cancer with a clinical perspective.  Nothing about K/P/A of south Asian immigrants. | PROQUEST CENTRAL/ NS |
|  | Betel Quid Use and Oral Cancer in a High-Risk Refugee Community in the USA: The Effectiveness of an Awareness Initiative | Shi, Lucy L.  Bradford, Ella  Depalo, Danielle E.  Chen, Amy Y.  2017 | Included | Study aims to investigate BN usage patterns, knowledge and the effectiveness of a visually guided educational initiative in a high-risk refugee population (Asians) in Georgia.  Two survey conducted among familiar and unfamiliar cohorts of 133 participants | PROQUEST CENTRAL/ NS |
|  | The impact of a community-based health education programme on oral cancer risk factor awareness among a Gujarati community | Siddique, I.  Mitchell, D. A.  2013 | Included | Aim was to determine any differences in oral cancer risk factor awareness and behaviours among first- and second-generation Gujarati Muslims and to investigate the impact of a community-based health education programme on oral cancer risk factor awareness.  (survey among 96 participants to check effect of education) | PROQUEST CENTRAL/ NS |
|  | Gutka and Tambaku Paan Use Among South Asian Immigrants: A Focus Group Study | Banerjee, Smita C.  Ostroff, Jamie S.  Bari, Sehrish  D'Agostino, Thomas A.  Khera, Mitali  Acharya, Sudha  Gany, Francesca  2014 | Included | Examination of (a) gutka and tambaku paan initiation and use patterns among South Asian immigrants, and (b) perceptions related to quitting and tobacco control.  Six focus groups were conducted- 39 adults included (Qualitative) | MANUAL SEARCH/RP |
|  | Awareness and use of South Asian tobacco products among South Asians in New Jersey | Hrywna, Mary  Lewis, M Jane  Mukherjea, Arnab  Banerjee, Smita C  Steinberg, Michael B  Delnevo, Cristine D  2016 | Included | Examination of the awareness and use of South Asian tobacco products as well as other potentially carcinogenic products such as supari, their context of use, and their cultural significance among South Asians living in the US.  Qualitative study – Eight -Focus groups and survey – 78 participants | MANUAL SEARCH/ RP |
|  | Oral cancer awareness in young South-Asian communities in London | Merchant, R.  Gallagher, J. E.  Scott, S. E.  2016 | Included | Cross sectional survey to evaluate knowledge and awareness of oral cancer amongst the young South-Asian community in London to determine whether demographic factors or health-related behaviours are associated with knowledge of oral cancer.  Cross- sectional questionnaire survey | MANUAL SEARCH/ RP |
|  | Betel quid chewing among Bangladeshi adolescents living in East London | Prabhu, NT  Warnakulasuriya, KAAS  Gelbier, S  Robinson, PG  2001 | Included | Cross sectional study to ascertain level and predictors of betel quid (pan) chewing in Bangladeshi adolescents.  Cross- sectional questionnaire | MANUAL SEARCH/ RP |
|  | The use of tobacco and betel quid ('pan') among Bangladeshi women in West Yorkshire | Summers, R. M.  Williams, S. A.  Curzon, M. E.  1994 | Included | Home based interviews using semi-structured questionnaires were undertaken among 296 first generation Bangladeshi women resident in West Yorkshire regarding pan chewing and associated risks.  Questionnaire and interviews | MANUAL SEARCH /RP |
|  | Health beliefs in oral cancer: Malaysian estate Indian scenario | Tan, Bee Siew  Ng, Kok Han  Esa, Rashidah  2001 | Excluded | Study to describe the health beliefs related to oral cancer (OC) in a high-risk group in Malaysia, a predominantly Indian community living in an agricultural setting called an estate.  Looking at feasibility of oral cancer- oral cancer screening, in estates in Malaysia (upper middle-income country) | MANUAL SEARCH/ RP |
|  | Knowledge, opinions, and practices related to oral cancer: Results of three elderly racial groups | Yellowitz, Janet A  Goodman, Harold S  Farooq, Naila S  1997 | Excluded | A study of 204 inner-city, senior center participants (ages ranged from 41-96years) was conducted to assess their knowledge, opinions, and practices related to oral cancer. (Participants were either White, African-American, or of Korean descent.)  Interview - questionnaire | MANUAL SEARCH /RP |
