# Supplementary material for: Knowledge, attitudes and practices of South Asian immigrants in developed countries regarding oral cancer: an integrative review
Source: BMC Cancer. 2020 May 27;20:477. doi: 10.1186/s12885-020-06944-9 (PMC7251750; doi:10.1186/s12885-020-06944-9)
Supplement: Supplementary file 5 — Additional file 5. Table of excluded studies. [file 12885_2020_6944_MOESM5_ESM.docx]

| cfvS.No. | Article topic | Authors  Year of Publication | Decision | Explanation | Database/Researcher |
| --- | --- | --- | --- | --- | --- |
|  | An assessment of oral cancer curricula in dental hygiene programmes: implications for cancer control | Thacker, K. K.  Kaste, L. M.  Homsi, K. D.  LeHew, C. W.  2016 | Excluded | Survey among faculty members of associate degree Dental hygiene programmes in Illinois, USA (related to oral cancer training components of their programme)  - Nothing specific about knowledge, attitudes and practices of South Asian immigrants regarding oral cancer risk. | CINAHL/ NS |
|  | Areca nut use following migration and its consequences | Warnakulasuriya, S.  2002 | Excluded | Review on available data on the prevalence of areca chewing migrant populations.  -Literature review | OVID MEDLINE/ NS |
|  | Assessing the oral cancer risk of South-Asian immigrants in New York City | Ahluwalia, K. P.  2005 | Excluded | Reference given study to be conducted in future to assess oral cancer related knowledge of south Asian adults in New York.  - Nothing specific about knowledge, attitudes and practices of South Asian immigrants regarding oral cancer risk. | SCOPUS/ NS |
|  | Areca nut and betel quid chewing among South Asian immigrants to Western countries and its implications for oral cancer screening | Auluck, A.  Hislop, G.  Poh, C.  Zhang, L.  Rosin, M. P  2009 | Excluded | Overview and discussion of the sociocultural beliefs, knowledge and practices regarding betel quid/areca nut chewing  - Literature review | SCOPUS/ NS |
| S.No. | Article topic | Authors  Year of Publication | Decision | Explanation | Database/Researcher |
|  | Areca nut and tobacco chewing habits in Durban, KwaZulu Natal | Bissessur, S.  Naidoo, S.  2009 | Excluded | Cross sectional study conducted South Africa (upper middle-income country) to investigate the prevalence of areca nut chewing, among migrant communities from India, Pakistan, and Dubai.  -doesn’t satisfy eligibility criteria | SCOPUS /NS |
|  | Oral cancer in Australia--risk factors and disease distribution | Cox, S.  2000 | Excluded | Overview of incidence of oral cancer and discussion on related practices and knowledge of south Asian immigrant communities.  - Literature review | SCOPUS/ NS |
|  | Betel quid chewing and the risk of oral and oropharyngeal cancers: A meta-analysis with implications for cancer control | Guha, N.  Warnakulasuriya, S.  Vlaanderen, J.  Straif, K.  2014 | Excluded | Meta-analysis of 50 publications assessing the relationship between oral/oropharyngeal cancer and chewing betel quid among Asians  -Nothing specific about knowledge, attitudes and practices of South Asian immigrants regarding oral cancer risk. | SCOPUS/ NS |
|  | Oral submucous fibrosis: Two cases of malignant transformation in asian immigrants to the United Kingdom | McGurk, M.  Craig, G. T.  1984 | Excluded | OSF cases among Asian immigrants in UK, are described.  Nothing specific about knowledge, attitudes and practices of South Asian immigrants regarding oral cancer risk. | SCOPUS / NS |
| S.No. | Article topic | Authors  Year of Publication | Decision | Explanation | Database/Researcher |
|  | Nasopharyngeal and hypopharyngeal carcinoma risk among immigrants in Sweden | Mousavi, S. M.  Sundquist, J.  Hemminki, K.  2010 | Excluded | High rates of hypo-pharyngeal carcinoma risk in immigrants (specifically Indian) in Sweden mentioned.  Family cancer database used  Nothing specific about knowledge, attitudes and practices of South Asian immigrants regarding oral cancer risk | SCOPUS/ NS |
|  | 'Betelmania' - Betel quid chewing by Cambodian women in the United States and its potential health effects | Pickwell, S. M.  Schimelpfening, S.  Palinkas, L. A.  1994 | Excluded | Survey undertaken of group of Cambodian refugee women.  Nothing specific about knowledge, attitudes and practices of South Asian immigrants regarding oral cancer risk | SCOPUS/ NS |
|  | Oral submucous fibrosis in a 31-year-old Indian woman. First case report from Germany | Reichart, P. A.  Philipsen, H. P.  2006 | Excluded | A case report of Indian woman with OSF in Germany (article full text not available in English)  Case report | SCOPUS/ NS |
|  | Evaluation of a culturally tailored smoking prevention program for Asian American youth | Ma, G. X.  Lan, Yajia  Edwards, R.L.  2004 | Excluded | This study evaluated effectiveness of a smoking prevention program for Asian American youth.  Nothing specific about knowledge, attitudes and practices of South Asian immigrants regarding oral cancer risk | SCOPUS/ NS |
|  | Oral Cancer Awareness among community-dwelling senior citizens in Illinois | Posorski, Ewa  Boyd, Linda  Giblin, Lori  Welch, Lisa  2014 | Excluded | The study assessed oral cancer awareness among senior citizen in Illinois.  Nothing specific about knowledge, attitudes and practices of South Asian immigrants regarding oral cancer risk | SCOPUS/ NS |
| S.No. | Article topic | Authors  Year of Publication | Decision | Explanation | Database/Researcher |
|  | Paan and Gutka in the United States: An Emerging Threat | Changrani, Jyotsna  Gany, Francesca  2005 | Excluded | Overview of Pan and gutka chewing among south Asian immigrants in USA.(pre-read article in series of next original article by same author)  Literature review | EMBASE/NS |
|  | Ethnicity and oral cancer | Scully, Crispian  Bedi, Raman  2000 | Excluded | Review article: Consideration of evidence of marked ethnic variations in the incidence, and survival of oral cancer.  Literature review | PROQUEST CENTRAL/ NS |
|  | Association of Betel Nut with Carcinogenesis: Revisit with a Clinical Perspective | Sharan, Rajeshwar N.  Mehrotra, Ravi  Choudhury, Yashmin  Asotra, Kamlesh  2012 | Excluded | Systematic review attempts to put in perspective the consequences of this widespread habit of BN/BQ mastication.  Nothing specific about knowledge, attitudes and practices of South Asian immigrants regarding oral cancer risk. | PROQUEST CENTRAL/ NS |
|  | Health beliefs in oral cancer: Malaysian estate Indian scenario | Tan, Bee Siew  Ng, Kok Han  Esa, Rashidah  2001 | Excluded | Looking at feasibility of oral cancer- oral cancer screening, in estates in Malaysia (upper middle-income country)  Doesn’t fit eligibility criteria | MANUAL SEARCH/ RP |
|  | Knowledge, opinions, and practices related to oral cancer: Results of three elderly racial groups | Yellowitz, Janet A  Goodman, Harold S  Farooq, Naila S  1997 | Excluded | A study of 204 inner-city, senior center participants conducted to assess their knowledge, opinions, and practices related to oral cancer.  (Participants were either White, African-American, or of Korean descent- not South Asians)  Nothing specific about knowledge, attitudes and practices of South Asian immigrants regarding oral cancer risk. | MANUAL SEARCH /RP |
