# Supplementary material for: Knowledge, attitudes and practices of South Asian immigrants in developed countries regarding oral cancer: an integrative review
Source: BMC Cancer. 2020 May 27;20:477. doi: 10.1186/s12885-020-06944-9 (PMC7251750; doi:10.1186/s12885-020-06944-9)
Supplement: Supplementary file 6 — Additional file 6. Critical appraisal of articles. [file 12885_2020_6944_MOESM6_ESM.docx]

| **Additional file 6: Critical appraisal of full text articles** | | | | | |
| --- | --- | --- | --- | --- | --- |
| S.No. | Author(year) | Article/paper | Methodology | Percentage (in %) | Quality |
|  | Summers et al  (1994) | The use of tobacco and betel quid (‘pan’) among Bangladeshi women in West Yorkshire | Quantitative | 62.5 | B |
|  | Pearson et al  (1999) | Dental service use and the implications for oral cancer screening in a sample of Bangladeshi adult medical care users living in Tower Hamlets, UK | Quantitative | 75 | B |
|  | Shetty et al  (1999) | Knowledge, attitudes and beliefs of adult South Asians living in London regarding risk factors and signs for oral cancer | Quantitative | 62.5 | B |
|  | Khan et al  (2000) | Predictors of tobacco and alcohol consumption and their relevance to oral cancer control amongst people from minority ethnic communities in the South Thames health region, England | Quantitative | 50 | B |
|  | Vora et al  (2000) | Alcohol, tobacco and paan use and understanding of oral cancer risk among Asian males in Leicester | Quantitative | 62.5 | B |
|  | Prabhu et al  (2001) | Betel quid chewing among Bangladeshi adolescents living in East London | Quantitative | 62.5 | B |
|  | Changrani et al (2006) | Paan and Gutka Use in the United States: A Pilot Study in Bangladeshi and Indian-Gujarati Immigrants in New York City | Quantitative | 50 | B |
|  | Croucher et al (2011) | Campaign awareness and oral cancer knowledge in UK resident adult Bangladeshi: a cross-sectional study | Quantitative | 75 | B |
|  | Lokhande et al (2013) | Chewing tobacco use among South-East Asian men in Auckland | Qualitative | 88.8 | A |
|  | Siddique et al  (2013) | The impact of a community-based health education  programme on oral cancer risk factor awareness among a Gujarati community | Quantitative | 62.5 | B |
|  | Banerjee et al (2014) | Gutka and Tambaku Paan Use Among South Asian Immigrants: A Focus Group Study | Qualitative | 88.8 | A |
|  | Hrywna et al  (2016) | Awareness and Use of South Asian Tobacco Products Among South Asians in New Jersey | Qualitative | 77.7 | B |
|  | Merchant et al  (2016) | Oral cancer awareness in young South-Asian communities in London | Quantitative | 50 | B |
|  | Shi et al  (2017) | Betel Quid Use and Oral Cancer in a High-Risk Refugee Community in the USA: The Effectiveness of an Awareness Initiative | Quantitative | 37.5 | C |
|  | Mukherjea et al (2018) | Moving Toward a True Depiction of Tobacco Behaviour Among Asian Indians in California: Prevalence and Factors Associated with Cultural Smokeless Tobacco Product Use | Quantitative | 37.5 | C |
|  | Petti et al  (2018) | Betel quid chewing among adult male immigrants from the Indian subcontinent to Italy | Quantitative | 75 | B |

Note: A or good quality (80-100%); B or fair quality (50-79%); C or poor quality (<50%)

Reference: Goldsmith MR, Bankhead CR, Austoker J. Synthesising quantitative and qualitative research in evidence-based patient information. Journal of Epidemiology & Community Health. 2007;61(3)
